# Genetic variability in multidrug-resistant *Mycobacterium tuberculosis* isolates from patients with pulmonary tuberculosis in North India

**DOI:** 10.1186/s12866-021-02174-6

**Published:** 2021-04-21

**Authors:** Ajay Vir Singh, Suman Singh, Anjali Yadav, Shweta Kushwah, Rajbala Yadav, Davuluri Kushma Sai, Devendra Singh Chauhan

**Affiliations:** Department of Microbiology and Molecular Biology, ICMR-National JALMA Institute for Leprosy and Other Mycobacterial Diseases, Agra, Uttar Pradesh 282004 India

**Keywords:** Spoligotyping, CAS1_DEL lineage, *Mycobacterium tuberculosis*, Multidrug resistant, North India

## Abstract

**Background:**

Information on the genetic variability of drug resistant isolates of *Mycobacterium tuberculosis* is of paramount importance to understand transmission dynamics of disease and to improve TB control strategies. Despite of largest number of multidrug-resistant (MDR) tuberculosis cases (1, 30,000; 27% of the global burden), strains responsible for the expansion or development of drug-resistant *Mycobacterium tuberculosis* infections have been poorly characterized in India. Present study was aimed to investigate the genetic diversity in MDR isolates of *Mycobacterium tuberculosis* in North India.

**Results:**

Spacer oligonucleotide typing (spoligotyping) was performed on 293 clinical MDR isolates of *Mycobacterium tuberculosis* recovered from cases of pulmonary tuberculosis from North India. Spoligotyping identified 74 distinct spoligotype patterns. Comparison with an international spoligotype database (spoldb4 database) showed that 240 (81.91%) and 32 (10.92%) strains displayed known and shared type patterns, while 21 (7.16%) strains displayed unique spoligotype patterns. Among the phylogeographic lineages, lineage 3 (East African-Indian) was found most predominant lineage (*n* = 159, 66.25%), followed by lineage 2 (East Asian; *n* = 34, 14.16%), lineage 1 (Indo-Oceanic; *n* = 30, 12.50%) and lineage 4 (Euro American; *n* = 17, 7.08%). Overall, CAS1_DEL (60.41%; SITs 2585, 26, 2694, 309, 381, 428, 1401, 141, 25, 1327) was found most pre-dominant spoligotype pattern followed by Beijing (14.16%; SITs255, 260, 1941, 269) and EAI3_IND (5.00%; SITs 298, 338, 11). The demographic and clinical characteristics were not found significantly associated with genotypic lineages of MDR-*M.tuberculosis* isolates recovered from pulmonary TB patients of North India.

**Conclusions:**

Present study reveals high genetic diversity among the *Mycobacterium tuberculosis* isolates and highlights that SIT141/CAS1_Del followed by SIT26/ Beijing lineage is the most common spoligotype responsible for the development and transmission of MDR-TB in North India. The high presence of shared type and unique spoligotype patterns of MDR strains indicates epidemiological significance of locally evolved strains in ongoing transmission of MDR-TB within this community which needs to be further monitored using robust molecular tools with high discriminatory power.

## Background

Tuberculosis (TB) remains as an important infectious disease and public health concern worldwide. The emergence of drug resistance in *M.tuberculosis* strains has further worsened the situation and presents major hurdle for effective management of disease in most of developed and under developed countries of the world including India. Multidrug resistant (MDR) TB has been defined as resistance to isoniazid and rifampicin with or without any other first line anti-TB drugs. Additional resistance to fluoroquinolones and second-line injectables has been considered as extensively drug-resistant (XDR) TB. It has been estimated that about 3.4% of new cases and 18% of previously treated cases of TB in the world have MDR-TB or rifampicin-resistant TB [1]. According to the World Health Organization (WHO) estimates, globally 4,84,000 cases of MDR/ rifampicin resistant TB were emerged and about 6.2% of MDR-TB cases were identified as XDR-TB in 2018 [1].

India has the highest number of TB patients in the world and is accountable for about 27% of new TB cases developed globally [1]. As per the report of the “First National Anti-Tuberculosis Drug Resistance Survey” more than 6% of TB patients in India have MDR-TB [2]. Recently WHO reported that the number of MDR-TB in India is 99,000 and the country is accounting for about one-fourth of the global burden of MDR-TB [1]. Despite the huge number of drug resistant TB patients, limited information is available on the genomic diversity of drug resistant strains of *M.tuberculosis* circulating in the country [3–6]. The genotypic diversity of MDR/XDR-TB isolates of *M.tuberculosis* in India needs to be investigated to better understand the transmission dynamics of drug resistant TB and to strengthen the activities of TB control program in the country. The advent of molecular methods such as Spacer oligonucleotide genotyping (spoligotyping), restriction IS6110-based fingerprinting and Mycobacterial interspersed repetitive units-variable number of tandem repeats (MIRU-VNTRs) has provided new ways to determine the genetic diversity and epidemiology of *M.tuberculosis* within the study population [7, 8]. Over the years, spoligotyping has been emerged as the most widely used molecular method for the investigation of genetic diversity and molecular epidemiology of *M. tuberculosis* in different countries including India [9–11]. Therefore, present study was aimed to identify predominant clades of spoligotypes responsible for the transmission and prevalence of MDR-TB in North India.

## Results

### Details of patient’s population and clinical MDR-TB isolates

The detail of all the 293 clinical MDR-*M.tuberculosis* included in this study (according to patient gender, age, living conditions and clinical characteristics) is summarized in Table 1. Out of the 293 isolates, 105 (35.83%) and 188 (64.16%) isolates belonged to female and male, respectively. The median age of the female and male patients was 24 years (range 18–72 years) and 33 years (range: 18–85 years), respectively. Geographically, the majority of isolates were from Uttar Pradesh (*n* = 285, 97.26%) and adjoining states (Bihar and Rajasthan) of North India. The drug resistance pattern of MDR-TB isolates in this study is summarized in Table 2. Out of the 293 MDR-*M.tuberculosis* isolates in this study 125 (42.66%) were resistant to all tested first line anti-TB drugs (Table 2).

**Table 1:** Clinical and socio-demographic characteristics of consecutively enrolled TB suspects

Variable	Category	Number	%
Gender	Female	105	35.83
	Male	188	64.16
Age	18–35	198	67.57
	36–55	68	23.20
	Above 55	27	9.21
Living conditions	Rural	95	32.42
	Urban	198	67.57
Weight loss	No	45	15.35
	Yes	248	84.64
Previous history of TB treatment	No	99	33.78
	Yes	194	66.21

**Table 2:** Drug resistance pattern of MDR-*M.tuberculosis* isolates (*n =* 293) and association with genotypic lineages

Drug resistance pattern	No. of strains (%)	No. of ***M. tuberculosis*** isolates in different genotypic lineages (%)				
		Lineage 1 (Indo-Oceanic)	Lineage 2 (East Asian)	Lineage 3 (East African-Indian)	Lineage 4 (Euro-American)	Un-clustered
MDR	293 (100)	30 (10.23)	34 (11.60)	159 (54.22)	17 (5.80)	53 (18.08)
MDR+ Ethambutol	157 (53.58)	12 (7.64)	23 (14.64)	85 (54.14)	11 (7.00)	26 (16.56)
MDR+ Streptomycin	167 (56.99)	18 (10.77)	25 (14.97)	90 (53.89)	8 (4.79)	26 (15.56)
MDR+ Ethambutol + Streptomycin	125 (42.66)	11(8.8)	21(16.8)	64 (51.2)	7 (5.60)	22(17.6)

### Genotypic variability among MDR *M.tuberculosis* isolates and distribution of lineages

A total of 293 clinical isolates of MDR-*M.tuberculosis* were analysed using spoligotyping and 74 spoligotype patterns were identified. The lineage distribution of the *M.tuberculosis* isolates in this study is shown in Table 2. Out of the 293 MDR- *M.tuberculosis* isolates, 240 (81.91%) isolates were clustered in 40 SITs in the SITVIT2 database (Table 3), while 32 (10.92%) isolates were shown shared type patterns by a clade or SIT number in SITVIT2 database (Table 4) and 21 (7.16%) isolates were identified with unique patterns (Table 5) making the total number of un-clustered isolates to be 53 (18.08%).

**Table 3:**
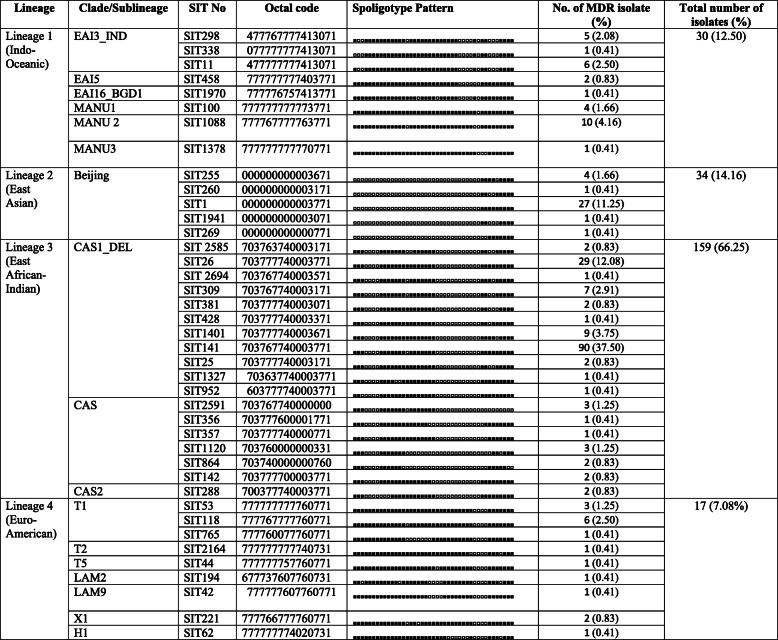
Genetic diversity of MDR-*Mycobacterium tuberculosis* isolates recovered from pulmonary TB patients in North India showed pre-existing type pattern (*n* = 240/293) in the SITVIT2 database

**Table 4:**
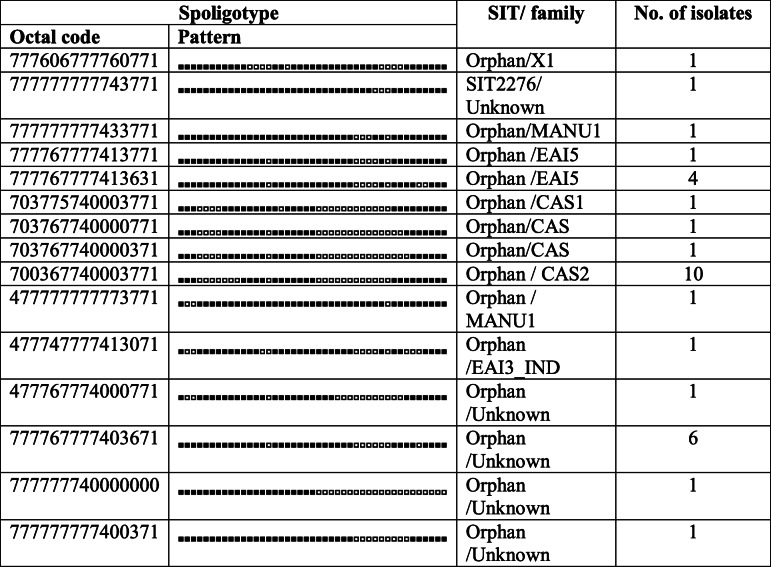
Description of 15 shared types containing 32 isolates of *M. tuberculosis* that matched a pre-existing shared type pattern in the SITVIT2 database

**Table 5:** Spoligotypes of 21 isolates not identified in SITVIT2 database by a SIT number or clade and analysed by SpotClust tool

Octal Code	No. of isolates	Probable family using SpotClust tool	Probability
477767777400071	1	EAI3	0.999
477767777403071	1	EAI3	0.999
777677776001730	1	EAI5	0.985
007777777403600	1	EAI5	0.997
373767777413731	1	EAI5	0.996
477001777403771	1	EAI5	0.999
477767777403771	1	EAI5	0.993
703767777403671	3	EAI5	0.990
777000777403771	1	EAI5	0.993
501767500002660	1	CAS	0.999
700367700001671	1	CAS	0.999
701767740003771	1	CAS	0.999
703601740003731	1	CAS	0.999
703764000003771	1	CAS	0.999
703767600003771	1	CAS	0.999
703767740000671	1	CAS	0.999
555747777643771	1	Family 33	0.999
557767777653771	1	Family 33	0.999
777667777620000	1	T2	0.999

Among the isolates of already defined spologotypes patterns (*n* = 240), East African-Indian was found to be most common lineage (*n* = 159, 66.25%) followed by East Asian (*n* = 34, 14.16%), Indo-Oceanic (*n* = 30, 12.50%) and Euro-American lineage (*n* = 17, 7.08%) in North India (Table 3). The majority of isolates belonged to CAS1_DEL (*n* = 145, 60.41%; SITs 2585, 26, 2694, 309, 381, 428, 1401, 141, 25, 1327) followed by Beijing (*n =* 34, 14.16%; SITs255, 260, 1941, 269) and EAI3_IND (*n* = 12, 5.00%; SITs 298, 338, 11) sub-lineages. The other minor sub-lineages includes MANU2 (*n*=10, 4.16%), MANU1 (*n*=4, 1.66%), MANU3 (*n*=1, 0.41%), T1(*n*=10, 4.16%), T2(*n*=1, 0.41%), T5(*n*=1, 0.41%), CAS (*n*=12, 5.00%), CAS2(*n*=2, 0.83%), X1 (*n*=2, 0. 83%), H1 (*n*=1, 0.41%), EAI16_BGD1 (*n*=1, 0.41%), LAM2 (*n*=1, 0.41%) and LAM9 (*n*=1, 0.41%) in present study (Table 3). The SIT141 and SIT26 of the CAS1/Del sub-lineage and SIT1of the Beijing sub-lineage were found predominant SITs in present study, all together accounting for 60.41% (145/240) of the clustered MDR-*M. tuberculosis* isolates (Table 3).

The isolates with a pre-existing shared type pattern (*n* = 32) hence were labelled as ‘orphan’, belonged to 15 distinct patterns of spoligotype (Table 4). Out of the 32 isolates, 23 (71.8%) were matched with a pre-existing SIT or clade in the SITVIT2 database while 9 isolates were matched with pre-existing ‘Orphan’ SIT and unknown clade pattern (Table 4). The isolates with unique spoligotype patterns (*n* = 21) were further analyzed to identify their most probable families using the SpotClust tool (Table 5). Five most probable families were recognized that included EAI5 with 9 (42.85%) isolates, CAS with 7 (33.33%) isolates, FAMILY-33 with 2 (9.52%) isolates, EAI3 with 2 (9.52%), and T2 with 1 (4.76%) isolates.

### Association of MDR-*M. tuberculosis* lineages with clinical and epidemiological features of the patients

The lineages of MDR-*M.tuberculosis* isolates were not found to be significantly associated with clinical and epidemiological features of the study population (Table 6). The clustered isolates were also not found to be significantly associated with clinical/epidemiological features of the patients (Table 7). The strains of predominant spoligo-type lineage (East African-Indian Lineage 3**)** was found higher in patients of male gender (*n* = 105, 66.03%), young age (18–35 years) group (*n* = 109, 68.55%), residents of rural areas (*n* = 63, 39.62%) and with the history of previous TB treatment (*n* = 110, 69.18%) as compared to other spoligotype lineages; but the effect was not statistically significant (Table 8).

**Table 6:** Distribution of MDR-*M.tuberculosis* isolates according to its genotypic lineages and clinical/epidemiological features of the patients

Variable	Category	Total no. of isolates (***n =*** 240)	No. of ***M. tuberculosis*** isolates in different genotypic lineages				***p***-value
			Indo-Oceanic	East Asian	East African-Indian	Euro-American	
Gender	Female	86	15	12	54	5	0.366
	Male	154	15	22	105	12	
Age	18–35	163	20	23	109	11	0.578
	36–55	54	9	6	36	3	
	Above 55	23	1	5	14	3	
Living conditions	Rural	95	11	13	63	7	0.987
	Urban	145	19	21	96	10	
Weight loss	No	39	5	5	26	3	0.993
	Yes	201	25	29	133	14	
Previous history of TB treatment	No	83	12	15	49	7	0.379
	Yes	157	18	19	110	10

**Table 7:** Association between clustered and un-clustered MDR-*M.tuberculosis* isolates with clinical/epidemiological features of the patients

Variable	Category	Total no. of isolates (***n =*** 293)	Clustered Isolates (%)	Un-clustered Isolates (%)	***p***-value
Gender	Female	105	86 (81.90)	19(18.09)	0.998
	Male	188	154 (81.91)	34 (18.08)	
Age	18–35	198	163 (82.32)	35 (17.67)	0.776
	36–55	68	54 (79.41)	14 (20.58)	
	Above 55	27	23 (85.18)	4 (14.81)	
Living conditions	Rural	110	95 (86.36)	15 (13.63)	0.124
	Urban	183	145 (79.23)	38 (20.76)	
Weight loss	No	45	39 (86.66)	6 (13.33)	0.367
	Yes	248	201 (81.04)	47 (18.95)	
Previous history of TB treatment	No	99	83 (83.83)	16 (16.16)	0.540
	Yes	194	157 (80.92)	37 (19.07)

**Table 8:** Association between the occurrence of most predominant genotypic lineage (East African-Indian) and clinical/epidemiological features of the patients

Variable	Category	Total no. of isolates (***n =*** 240)	East African-Indian Isolates (***n =*** 159) (%)	Others genotypic lineage (***n*** = 81) (%)	***p***-value
Gender	Female	86	54 (33.96)	32 (39.50)	0.397
	Male	154	105 (66.03)	49 (60.49)	
Age	18–35	163	109 (68.55)	54 (66.66)	0.847
	36–55	54	36 (22.64)	18 (22.22)	
	Above 55	23	14 (8.80)	9 (11.11)	
Living conditions	Rural	95	63 (39.62)	32 (39.50)	0.986
	Urban	145	96 (60.37)	49 (60.49)	
Weight loss	No	39	26 (16.35)	13 (16.04)	0.952
	Yes	201	133 (83.64)	68 (83.95)	
Previous history of TB treatment	No	83	49 (30.81)	34 (41.97)	0.0857
	Yes	157	110 (69.18)	47 (58.02)	

## Discussion

Despite the largest number of MDR-TB patients (99,000 cases; 27% of global cases), less is known about the genetic biodiversity of MDR-*M.tuberculosis* strains in India. Most of the studies done so far either explored only the circulating genotypes of *M. tuberculosis* or included less number of MDR-*M.tuberculosis* isolates in the country [3–6, 12, 13]. To our knowledge, present study includes largest number of clinical MDR *M.tuberculosis* isolates (*n* = 293) to describe the predominant lineages and sub-lineages circulating in pulmonary MDR-TB patients in North India. In present study, high genotypic diversity (74 spoligotype patterns) was observed and we found that majority (81.91%) of MDR-isolates were clustered into 40 spoligotype international types (SITs) in the SITVIT2 database. Similarly, Diriba et al., [14] investigated genetic diversity in MDR *M.tuberculosis* isolates in Ethiopia and identified 43 spoligotype patterns and high proportion of clustering (86.0%) in MDR-TB isolates. In India, Desikan et al., [15] investigated the genetic diversity of *M. tuebrculosis* in TB patients from central India and reported the clustering of 70.3% isolates into 25 SITs. Chawla et al., [5] reported the clustering of 51.35% *M.tuberculosis* isolates into 11 SITs in patients from South India. In present study, the variation in clustering rate of isolates may be due to the inclusion of only MDR isolates of *M. tuberculosis*, difference in study period and/or geographical variation. The strains of shared type pattern (orphan) and without an assigned spoligotype (unique), made up 18.08% (53/293) of the total isolates. The epidemiological significance of these strains needs further monitoring using advanced clinical and molecular tools with better discriminatory power.

In present study, four major lineages of *M.tuberculosis* were identified; East African-Indian lineage 3 (54.26%) was found to be the most prevalent lineage followed by East Asian lineage 2 (11.60%), Indo-Oceanic lineage 1 (10.23%) and Euro-American lineage 4 (5.80%) in North India. Considering the genotype patterns, current study confirms the occurrence of complex genetic diversity of MDR-TB in north India which ranges from ancient lineage (lineage 1) to modern TB lineages (lineage 2, 3 and 4) of *M. tuberculosis*. The higher clustering rate of the strains in modern lineages (88.08%) as compared to ancient lineage (11.91%) in our study indicates the substantial recent ongoing transmission of drug resistant-TB in North India. The strains of East African-Indian lineage 3 have supposed to be linked with lower virulence, drug resistance and ability to spread [16]. Therefore the environmental and biological factors linked to enhanced levels of drug resistance and likely to expediting their expansion in particular geographical region need to be addressed.

Considering the spoligotyping patterns, the majority of the isolates in present study were clustered in CAS1_Del family (*n* = 148, 50.51%) followed by Beijing (*n* = 33, 11.26%). Similar to the present study, Sharaf-Eldin et al., [17] reported that the *M. tuberculosis* strains of CAS1_Delhi lineage were more likely to develop drug resistance or MDR compared to those with other spoligotype patterns in Sudan. In present study, Beijing genotype; which is supposed to be widespread and considered as a predilection for the development of drug resistance in *M. tuberculosis* strains was found as second most prevalent genotype in MDR strains in North India. Previously, Arora et al., [18] reported high proportion of Beijing (41.81%) as compared to CAS (36.36%) genotypes among MDR strains from paediatric TB patients from Delhi, India. Almeida et al., [4] also reported a high frequency of the Beijing strains (35%) as compared to Delhi genotype (31%) among MDR isolates recovered in and around Mumbai, India. Previously, it has been noted that the association between Beijing strains and MDR varies worldwide [19, 20]. The difference between the findings of present study and earlier studies from India may be attributed to the variation in the predominant genotypes in study areas, difference in study duration and variation in sample size and/ or study designs. Recently, Desikan et al., [15] investigated the predominant circulating genotypes of *M. tuberculosis* in Bhopal, Central India and identified CAS1_DEL followed by EAI3_IND as the most predominant type.

In present study, SIT141 (*n* = 96; 32.76%) and SIT26 (*n* = 28; 9.55%) of CAS1_Del family followed by SIT1 (*n* = 27; 9.21%) of Beijing family were found the predominant SITs among MDR-TB isolates in North India. Previously, the presence of SIT141/CAS1_Del has also been identified in strains from Delhi and different regions of the country [4, 18, 21]. The presence of SIT26/CAS1-Delhi has been mainly reported from countries of the Middle-East and Central Asia, or regions that have witnessed an important migration to or from the Indian sub-continent, e.g. Africa, East Asia, United States and Europe [22, 23]. Recently, Desikan et al., [15] reported the predominance of SIT26/CAS1_Del strains in central India. Basir et al., [13] also reported the predominance of SIT26/ CAS1_Del in isolates from Kashmir Valley, India. Arora et al. [18] reported SIT1/Beijing and SIT26/CAS1-Delhi as predominant genotype among the XDR-TB isolates in and around the Delhi region of India. The predominance of CAS1-Delhi and Beijing strains among MDR-TB patients in this study and presence of these clades in other parts of the country [5, 12, 13, 24] underlines the rapid dissemination of these lineages across large geographical regions in India. In present study, Beijing clade is followed by ill-defined Manu (5.11%), EAI3_IND clade (4.09%), T (4.09%), CAS (3.41%), CAS2 (0.68%), LAM (0.68%), X (0.68%) and Haarlem (0.34%) in decreasing order. These observations indicated the complex diversity of circulating *M.tuberculosis* strains among MDR –TB patients and reflect the occurrence of different transmission pathways for MDR-TB in North India.

In present study, no statistically significant association was found between the occurrence of genotypic lineages of MDR-*M.tuberculosis* isolates and clinical or epidemiological features of the study population. Similar to the present study, Desikan et al., [15] also did not find any association between clustered isolates and demographic variables like age, and gender and treatment history of the TB patients in central India. Similarly, Niobe-Eyangoh et al., [25] did not find any significant association between genotypic families of *M.tuberculosis* complex isolates form pulmonary TB patients and patient characteristics (sex, age, and human immunodeficiency virus status) in Cameroon. However, Jiao et al., [26] investigated the strain diversity of *M. tuberculosis* isolates from pediatric cases and found that the prevalence rate of Beijing family strains were significantly higher (*P* value = 0.029) in new cases (91.0%) as compared to previously treated cases (69.6%) among  children in China. The explanation for the differences may be due to the geographical variation, difference in predominant genotypic lineage of *M. tuberculosis,* sample size and methodology of the study. Further attention is needed to understand the clinical, genetic and social correlations of the evolution of drug resistant *M. tuberculosis* strains in North India.

## Conclusions

In conclusion, our study highlights that the MDR-TB in North India is caused due to heterogeneous groups predominated by CAS1_Delhi (SIT141 and 26) and Beijing (SIT1) lineages of *M. tuberculosis*. The higher presence of orphan and unique spoligotype patterns among MDR isolates in present study indicates high evolutionary pressure and clonal expansion of locally evolved strains in North India, which may have the potential to be a growing threat to public health. The result of this study emphasise the need of continuous surveillance of genetic diversity among drug resistant especially MDR and XDR-TB strains of *M. tuberculosis* to better understand the evaluation and transmission dynamics of drug resistant TB in India.

## Methods

The study was carried out from January 2017 to January 2020 in the department of Microbiology and Molecular Biology, ICMR-National JALMA Institute for Leprosy and other Mycobacterial Diseases, India. All experiments and methods were performed in accordance with relevant guidelines and regulations. All the study protocols were approved by the Institutional Human Ethics Committee.

### *Mycobacterium tuberculosis* isolates

During the period of 2017–2019, a total of 293 MDR isolates were recovered from sputum samples of suspected MDR-TB cases and included in this study. After appropriate counseling and written informed consent; detailed demographic information, clinical history and physical characteristics of all the study participants were recorded. The sputum samples of the study participants were processed for the isolation of mycobacteria on Lowenstein–Jensen medium as per the method described by Raizada et al., [27]. The species level identification of the isolates was performed using standard biochemical tests viz. nitrate reduction, catalase activity at 68 °C, Tween 80 hydrolysis, aryl sulphatase test and growth on Mac’Conkeys medium as per CDC manual given by Vestal [28]. The drug susceptibility patterns of *M.tuberculosis* isolates were studied against first line anti-TB drugs (rifampicin, isoniazid, ethambutol and streptomycin) using standard minimum inhibitory concentration method on LJ-medium as per the guideline of revised national tuberculosis control program, India. All the microbiological tests were performed in Biosafety level-3 (BSL-3) laboratory, Department of Microbiology and Molecular Biology, NJIL&OMD, Agra.

### DNA extraction

DNA was extracted from the log phase growth of *M. tuberculosis* isolates using the physical- chemical and enzymatic method [29]. Briefly, about 2 loopful growth of *M. tuberculosis* was suspended in 400 μl of TE buffer and heated for 15 min at 95 °C followed by immediate chilling in ice for 15 min. The step was repeated thrice. To this 40 μl lysozyme (20 μg/ml) was added, mixed gently and incubated for 2 h at 37 °C. After this, 56 μl of 10% SDS was added and mixed gently by inverting the tubes 5–6 times followed by addition of 5.0 μl of proteinase K (10 mg/ml) and mixed well using vortex. Mixture was incubated at 65 °C for 30 min. After incubation, 80 μl of 5 M NaCl and 64 μl of pre-warmed CTAB / NaCl solution was added and milky tube content was mixed well and incubated at 65 °C for 30 min. Equal volume of freshly prepared chloroform - isoamyl alcohol (24:1) mixture was added and vortexed for mixing and centrifuged at 10000 rpm for 5 min. After centrifugation, three layers became visible; upper (aqueous) layer (about 300 μl) was transferred into a new sterile eppendrof tube. To the aqueous layer, 0.7 volume of iso-propanol alcohal (180 μl) was added and gently mixed and incubated at -20 °C overnight allowing DNA precipitation. Next day the solution was centrifuged at 10000 rpm for 15 min, supernatant was discarded and sediment was washed with 150 μl of 70% chilled ethanol by centrifugation at 10000 rpm for 5 min. The supernatant was carefully discarded without disturbing the pellet. The washing step was repeated, the tube was allowed to air dry. Dried pellet was re-suspended in 30 μl of TE buffer and stored at -20 °C for further use in spoligotyping.

### Spoligotyping and analysis of patterns

Spoligotyping was carried out by amplifying the whole DR region using the commercially available kit (Mapmygenome, Hyderabad) as per the method previously described by Sharma et al., [9]. Briefly, the extracted genomic DNA of *M.tuberculosis* isolates was subjected to PCR to amplify direct repeats (DR region) and interspersed known spacers region using primers designated as DRa and DRb (Mapmygenome, Hyderabad). The PCR product was hybridized to 43 covalently bound oligonucleotides derived from the spacer sequence of *M. tuberculosis* H37Rv and *M. bovis* BCG. Hybridization signals were recorded by enhanced chemiluminescence detection system by exposing ECL-Hyper film (Amersham, GE Health Care). Distilled water was used as negative control and H37Rv and *M.bovis* BCG were used as positive controls. The presence and absence of spacer oligonucleotides were documented in the form of binary code that was converted into octal code and was compared with the international Database SITVIT WEB2. Spoligo patterns not found in SITVIT WEB2 were analyzed using “Spotclust”.

### Association of spoligotype lineages and epidemiologic characteristics of the patient’s

The association between demographic information, clinical / epidemiological data of the patients and spoligotyping results of MDR-*M.tuberculosis* isolates were analysed. For the comparison of categorical variables, significance testing was performed by χ2 test by 2-sided Fisher exact test as appropriate. The criterion for significance was set at *P* < 0.05 based on a two-sided test. Collected data were computerized using Excel, cleaned, and entered to be analyzed using STATA software.

## Data Availability

The datasets used and analysed in present study are available from the corresponding author on reasonable request.

## Abbreviations

*M.tuberculosis*: *Mycobacterium tuberculosis*
MDR: Multidrug-resistant
SIT: Spoligotype international types
TB: Tuberculosis
XDR: Extensively drug-resistant
WHO: World Health Organization
